# Obesity and Association with Area of Residence, Gender and Socio-Economic Factors in Algerian and Tunisian Adults

**DOI:** 10.1371/journal.pone.0075640

**Published:** 2013-10-08

**Authors:** Madjid Atek, Pierre Traissac, Jalila El Ati, Youcef Laid, Hajer Aounallah-Skhiri, Sabrina Eymard-Duvernay, Nadia Mézimèche, Souha Bougatef, Chiraz Béji, Leila Boutekdjiret, Yves Martin-Prével, Hassiba Lebcir, Agnès Gartner, Patrick Kolsteren, Francis Delpeuch, Habiba Ben Romdhane, Bernard Maire

**Affiliations:** 1 INSP (National Institute of Public Health), Algiers, Algeria; 2 IRD (Institut de Recherche pour le Développement), NUTRIPASS Research Unit, Montpellier, France; 3 INNTA (National Institute of Nutrition and Food Technology), Tunis, Tunisia; 4 SURVEN (Nutrition Surveillance and Epidemiology in Tunisia) Research Unit, Tunis, Tunisia; 5 INSP (National Institute of Public Health), Tunis, Tunisia; 6 Faculty of Medicine, El Manar University, Tunis, Tunisia; 7 ITM (Institute of Tropical Medicine), Antwerp, Belgium; 8 Epidemiology and Prevention of Cardiovascular Diseases Unit, Faculty of Medicine, Tunis, Tunisia; University of Bristol, United Kingdom

## Abstract

**Introduction:**

The epidemiological transition has resulted in a major increase in the prevalence of obesity in North Africa. This study investigated differences in obesity and its association with area of residence, gender and socio-economic position among adults in Algeria and Tunisia, two countries with socio-economic and socio-cultural similarities.

**Methods:**

Cross-sectional studies used stratified, three-level, clustered samples of 35–70 year old adults in Algeria, (women n = 2741, men n = 2004) and Tunisia (women n = 2964, men n = 2379). Thinness was defined as Body Mass Index (BMI) = weight/height <18.5 kg/m^2^, obesity as BMI ≥30, and abdominal obesity as waist circumference/height ≥0.6. Associations with area of residence, gender, age, education, profession and household welfare were assessed.

**Results:**

Prevalence of thinness was very low except among men in Algeria (7.3% C.I.[5.9–8.7]). Prevalence of obesity among women was high in Algeria (30.1% C.I.[27.8–32.4]) and Tunisia (37.0% C.I.[34.4–39.6]). It was less so among men (9.1% C.I.[7.1–11.0] and 13.3% C.I.[11.2–15.4]).The results were similar for abdominal obesity. In both countries women were much more obesity-prone than men: the women versus men obesity Odds-Ratio was 4.3 C.I.[3.4–5.5] in Algeria and 3.8 C.I.[3.1–4.7] in Tunisia. Obesity was more prevalent in urban versus rural areas in Tunisia, but not in Algeria (e.g. for women, urban versus rural Odds-Ratio was 2.4 C.I.[1.9–3.1] in Tunisia and only 1.2 C.I.[1.0–5.5] in Algeria). Obesity increased with household welfare, but more markedly in Tunisia, especially among women. Nevertheless, in both countries, even in the lowest quintile of welfare, a fifth of the women were obese.

**Conclusion:**

The prevention of obesity, especially in women, is a public health issue in both countries, but there were differences in the patterning of obesity according to area of residence and socio-economic position. These specificities must be taken into account in the management of obesity inequalities.

## Introduction

As a consequence of globalization and major socio-economic and demographic changes in recent decades, the epidemiological transition has resulted in a growing burden of non-communicable diseases (NCD) in low and middle income countries (LMIC) [1]. South Mediterranean countries have been particularly affected and overweight and obesity are now major public health issues in the region [2]. Intergenerational causes interacting with lifestyle factors such as dietary changes or lower levels of physical activity are well documented risk factors of obesity [1], . However, most of these lifestyle factors are determined by environmental or socio-economic factors, meaning that the analysis of the environmental and socio-economic patterning of obesity is essential for prevention [4], [5], [6].

Similarities between South Mediterranean countries in ethnicity, socio-cultural context and their mid-level development might predict similarities in both corpulence and its relationship with environmental and socio-economic factors [7]. However, if Maghreb countries do share a common socio-cultural background, even within such an apparently homogeneous area there is variability regarding socio-cultural factors [8], [9], [10]. Also, despite similarities, neighboring countries such as Algeria and Tunisia have followed somewhat different paths of socio-economic development during the last decades, e.g. Tunisia has a more diversified economy than Algeria with its dependence on the hydrocarbon rent [11]. Studies on obesity and its association with socio-economic factors among adults in Maghreb countries do exist but their comparability is hindered by different time periods, gender, targeted age-groups, or methodology [7], [12], [13]. Meaningful comparisons between countries ideally require standardized and comparable data such as Demographic and Health Surveys (DHS), which have been extensively used for this purpose in LMIC [5], [14]. They are nevertheless not ideal for all purposes (e.g. they often only focus on women) and no such data exists for Algeria [14] nor does recent data for Tunisia [15]. To document the nutrition transition and associated factors in North Africa, a research project implemented two national surveys in Algeria and Tunisia at the same time period with analogous methodologies. The surveys were planned collaboratively by a multinational team, including Algerian and Tunisian researchers who collaborated closely on the design of the survey, and developed the questionnaire and data collection procedures together.

The objective of this study was thus to use these surveys to assess similarities and differences in anthropometric status and investigate the association with urban versus rural area of residence, gender, and several dimensions of socio-economic position among adults in Algeria compared to Tunisia.

## Methods and Procedures

### Study Area

Algeria and Tunisia are neighboring South Mediterranean countries. By area, Algeria is the largest country in Africa compared to Tunisia, which is 15 times smaller, and the population comparison is 33 to 10 million inhabitants respectively. Both populations are about two-thirds urban and are at a middle level of development: Tunisia is 91^st^ out of 177 countries on the Human Development Index and Algeria 104^th^ [8].

### Survey Design and Subjects

In both countries, the target population was 35–70 year-old adults, which were deemed to present a more altered health status in relation to the long term effect of NCDs risk factors. Also, this age class was used in a number of studies pertaining to risk factors of chronic diseases [16]. Cross-sectional surveys used three-stage random cluster samples [17]. In Algeria the survey took place in June and July 2005. Sixteen wilayas (administrative divisions) out of 48 were randomly selected, and then 126 census districts were randomly selected with a probability proportional to the number of eligible households. Forty households were sampled in each district, and finally, one person was randomly selected in each household in the list resulting from the enumeration of all household members. In Tunisia, the survey took place from April to September 2005. Based on the 2004 census, a similar three-stage random clustered sample was stratified according to the seven administrative regions. Forty-seven census districts were randomly selected in each region, with a probability proportional to the number of eligible households and 20 households were sampled in each district. Finally in each household, one subject was selected at random from the enumeration of eligible subjects.

### Measurements

#### Area of residence, socio-economic and demographic variables

The urban-rural classification of districts was determined according to the national statistical institutes in each country. In Tunisia, urban districts were those belonging to large cities (≥100 000 inhabitants) and other cities (<100 000 inhabitants) with the definition of a city being that it featured a “municipality” i.e. an administrative structure. Rural districts were those featuring either grouped habitats (>80 households) or areas featuring dispersed habitats or groups in small villages. In Algeria, urban districts were those in “agglomerations” i.e. groups of at least 100 constructions with less than 200 meters between one another, and rural districts were those from “dispersed habitat” areas with small (<100) groups of constructions, hamlets, or dispersed habitats. Data on age, gender, and marital status was collected. Several dimensions of socio-economic position (SEP) were taken into account such as education, profession and household welfare level [6]. For the education and the profession of the subject, the last school class attended by the subject and the professional category were collected based on detailed classifications from the national statistical institutes in each country. For analysis purposes, they were then recoded into three categories of education and profession **(** **Table 1** **)**. Separately for each country, an asset-based index of household welfare was derived by multiple correspondence analysis of a set of items pertaining to housing characteristics and ownership of appliances. A detailed analysis of the relationships between the items and the first principal component was then performed, which enabled its interpretation as a continuous gradient of wealth (material living conditions). For each household, the value of the component is a weighted average of the binary variables coding for the different items, which has no absolute meaning (so that the value itself has no direct interpretation). But such an index can be used for ranking the households according to increasing level of welfare either using the continuous index itself and/or as a categorical variable after recoding. For analysis purposes this continuous index was recoded into quintiles of increasing welfare [6], [12], [18].

**Table 1:** Area of residence and socio-economic factors among 35–70 year old Algerian and Tunisian adults, by gender.

	Algeria (n = 4746)				Tunisia (n = 5343)		
	%^a^				%^a^		
	Women	Men	W vs. M^b^	Women		Men	W vs. M^b^
**Area**	n = 2742	n = 2004		n = 2964		n = 2379	
Urban	65.5	61.1	P = 0.18	66.6		68.3	P = 0.32
Rural	34.5	38.9		33.4		31.7	
**Age**	n = 2742	n = 2004		n = 2964		n = 2379	
35–44 y.	35.7	32.3		42.4		42.8	
45–54 y.	31.8	28.4	P = 0.0028	31.6		30.7	P = 0.86
55–70 y.	32.5	39.3		26.0		26.5	
**Marital status**	n = 2741	n = 2003		n = 2963		n = 2366	
Single	5.1	4.9		4.8		2.5	
Married	77.6	92.9	P<0.0001	81.0		94.2	<0.0001
Divorced/widowed	17.3	2.2		14.2		3.3	
**Education**	n = 2738	n = 2000		n = 2963		n = 2378	
No formal schooling	53.5	32.4		48.9		20.6	
Primary school	24.8	31.4	P<0.0001	31.7		38.4	P<0.0001
Secondary or more	21.7	36.2		19.4		41.0	
**Professional activity**	n = 2734	n = 1998		n = 2963		n = 2378	
Not working/retired	89.9	38.3		76.2		18.7	
Employee/worker	5.2	38.9	P<0.0001	15.9		56.8	P<0.0001
Upper/Intermediate	4.9	22.8		7.9		24.5	
**Household welfare index** c	n = 2691	n = 1964		n = 2805		n = 2254	
First quintile	17.5	21.1		21.6		19.0	
Second quintile	18.7	21.5		21.1		18.6	
Third quintile	19.4	20.0	P = 0.018	20.4		20.0	P = 0.012
Fourth quintile	23.8	21.1		17.7		21.3	
Fifth quintile	20.6	16.3		19.2		21.2	

a Weighted proportions.

b Null hypothesis of identical distribution in women vs. men (P-value adjusted for sampling design).

c Asset-based household welfare index: increasing welfare from 1^st^ to 5^th^ quintile.

#### Anthropometry

Standing height was measured to the nearest mm using a wall-mounted stadiometer (Person-check®, Kirchner & Wilhelm, Germany). Weight (with light clothing) was measured to the nearest 100 g on a calibrated scale (Detecto, USA). Waist circumference (WC) was measured with a flexible steel tape at the midpoint between the lower rib and the iliac crest to the nearest mm [19]. Overall adiposity was assessed by body mass index (BMI) = weight/height^2^. Thinness was defined as BMI <18.5 kg/m^2^, overweight (including obesity) as BMI ≥25.0 and obesity as BMI ≥30 [20]. Abdominal adiposity was assessed using the waist-to-height ratio (WHtR) and for both men and women the same ≥0.50 and ≥0.60 thresholds defined increasing levels of abdominal adiposity [21], [22].

#### Data collection

Surveys were conducted during home visits by trained field workers using standardized anthropometric measurements and socio-demographic questionnaires.

### Data Management and Statistical Analysis

Epidata software version 3.1 was used for data entry and validation and Stata 12 for data management and analysis [23], [24]. For continuous anthropometric response variables, linear models [25] estimated associations by crude and adjusted means and/or differences in means. For binary anthropometric response variables, logistic regression models were used to assess crude and adjusted prevalence odds-ratios (OR) and/or prevalence proportions [26]. Except for the specific issue of within country gender contrasts, relationships of obesity with area and socio-economic factors were analyzed in each country separately for each gender, so as to compare and discuss the measures of association “between countries within gender”. The type I error risk was set at 0.05. Results are given as estimates and design based standard errors (in parentheses) and/or 0.95 confidence intervals (in brackets). All analyses took the sampling design (stratification, clustering and sampling weights) into account [27] using *svy* Stata commands.

### Ethics

The surveys in both countries were carried out in accordance with the ethical principles for medical research involving human subjects. The subjects in both countries gave their free informed consent (verbally when it was not possible otherwise, e.g. in the case of illiteracy). Data was analyzed anonymously. In Algeria, the survey protocol was approved and authorized by the National Statistical Office. In Tunisia, the survey protocol was approved by the Ethics Committee on Human Research of the National Institute of Nutrition and the National Council of Statistics.

## Results

### General Characteristics of the Subjects

After accounting for refusals, absence, missing or outlying anthropometric values, 4746 subjects in Algeria and 5343 in Tunisia were analyzed **(** **Table 1** **)**, thus resulting in an overall response rate of 94.1% and 81.2% respectively. The response rates were lower among men than among women in both Algeria (79.5% versus 94.4% respectively) and Tunisia (72.3% versus 90.1% respectively). The response rates were also slightly lower in urban areas and/or more developed regions especially in Tunisia (detailed data not shown).

Within-gender distributions of marital status were similar in the two countries. The level of education was somewhat higher in Tunisia both overall and by gender. In both countries, the majority of women did not work outside the home, even more so in Algeria, where also more than one-third of the men did not work outside the home **(** **Table 1** **)**.

### Anthropometric Status by Gender and Country

For women, in both Algeria and Tunisia respectively the national prevalence of overweight (66.5%[63.7–69.4] and 71.1%[68.5–73.6]), of obesity (30.1%[27.8–32.5] and 37.0%[34.5–39.6]) and of abdominal obesity (30.9%[28.2–33.5] and 42.6%[39.8–45.4]) were very high, all the more in Tunisia **(** **Table 2** **)**. The prevalence of extreme obesity (BMI≥40) was around 2% in both countries. For women, the prevalence of thinness was very low in Tunisia, less so in Algeria. For men, thinness was not negligible in Algeria (7.3%[5.9–8.7]) but much less prevalent in Tunisia (3.2%[2.2–4.2]). The prevalence of overweight was high among men in both countries (Algeria 41.3%[38.5–44.0] and Tunisia 51.7%[48.2–55.1]). Among men, the prevalence of obesity was milder, either regarding overall obesity (Algeria 9.1%[7.1–11.0] and Tunisia 13.3%[11.2–15.4]) or abdominal obesity as WHtR> = 0.6 (Algeria 13.4%[11.2–15.6] and Tunisia 15.6%[13.5–17.8]). For men also, the rates of overweight and obesity were somewhat higher in Tunisia compared to Algeria.

**Table 2:** Anthropometric characteristics of 35–70 year old Algerian and Tunisian adults by gender and area.

	Algeria (n = 4746)							Tunisia (n = 5343)						
	All		Urban		Rural		U vs. R	All		Urban		Rural		U vs. R
	Meanor %^a^	s.e.^b^	Meanor %^a^	s.e.^b^	Meanor %^a^	s.e.^b^	P^c^	Meanor %^a^	s.e.^b^	Meanor %^a^	s.e.^b^	Meanor %^a^	s.e.^b^	P^c^
**Women**	n = 2742		n = 1735		n = 1007			n = 2964		n = 1638		n = 1326		
**Basic characteristics**														
Weight (kg)	68.6	0.3	69.7	0.5	66.4	0.6	<0.0001	69.4	0.4	71.8	0.5	64.7	0.6	<0.0001
Height (cm)	158.1	0.2	158.6	0.3	157.2	0.5	0.016	156.4	0.2	156.6	0.2	156.2	0.2	0.25
Waist circumference (cm)	88.5	0.5	89.5	0.6	86.8	0.7	0.005	91.2	0.4	93.1	0.5	87.4	0.7	<0.0001
**Overall adiposity**														
Body Mass Index (kg/m^2^)	27.4	0.2	27.7	0.2	26.9	0.2	0.006	28.4	0.2	29.3	0.2	26.5	0.2	<0.0001
Thinness (BMI <18.5)	3.6%	0.4	3.1%	0.5	4.6%	0.8	0.096	1.8%	0.3	0.8%	0.2	3.9%	0.7	<0.0001
Overweight (BMI ≥25.0)	66.5%	1.4	68.7%	1.8	62.3%	2.2	0.027	71.1%	1.3	77.8%	1.6	57.7%	2.0	<0.0001
Obesity (BMI ≥30.0)	30.1%	1.2	31.7%	1.5	27.1%	1.6	0.046	37.0%	1.3	43.7%	1.6	23.7%	1.8	<0.0001
Extreme obesity (BMI ≥40.0)	1.7%	0.3	2.2%	0.4	0.8%	0.3	0.007	2.3%	0.3	2.7%	0.5	1.4%	0.4	0.028
**Abdominal adiposity**														
Waist to height ratio × 100	56.0	0.3	56.5	0.4	55.3	0.4	0.037	58.4	0.2	59.6	0.3	56.0	0.4	<0.0001
Waist to height ratio≥0.50	75.5%	1.4	77.1%	1.8	72.6%	2.0	0.12	82.4%	1.0	87.2%	1.1	72.7%	2.0	<0.0001
Waist to height ratio≥0.60	30.9%	1.3	32.7%	1.7	27.4%	2.3	0.069	42.6%	1.4	47.6%	1.8	32.6%	2.2	<0.0001
**Men**	n = 2004		n = 1185		n = 819			n = 2379		n = 1423		n = 956		
**Basic characteristics**														
Weight (kg)	71.1	0.5	71.7	0.6	70.1	0.9	0.12	73.6	0.5	75.3	0.6	69.9	0.6	<0.0001
Height (cm)	170.9	0.3	170.7	0.3	171.2	0.5	0.40	170.2	0.2	170.3	0.3	169.9	0.3	0.28
Waist circumference (cm)	88.0	0.6	88.6	0.8	87.1	0.8	0.19	91.0	0.4	92.5	0.5	87.6	0.5	<0.0001
**Overall adiposity**														
Body Mass Index (kg/m^2^)	24.3	0.2	24.6	0.2	23.9	0.3	0.020	25.3	0.1	25.9	0.2	24.2	0.2	<0.0001
Thinness (BMI <18.5)	7.3%	0.7	6.3%	0.9	8.9%	1.1	0.065	3.2%	0.5	1.9%	0.4	6.0%	1.3	0.0029
Overweight (BMI ≥25.0)	41.3%	1.4	44.1%	1.8	36.9%	2.2	0.012	51.7%	1.8	57.7%	2.2	38.6%	2.2	<0.0001
Obesity (BMI ≥30.0)	9.1%	1.0	8.6%	1.1	9.8%	2.0	0.58	13.3%	1.1	15.0%	1.5	9.7%	1.3	0.0073
Extreme obesity (BMI ≥40.0)	0.4%	0.1	0.3%	0.2	0.5%	0.2	0.35	0.6%	0.3	0.8%	0.4	0.2%	0.1	0.13
**Abdominal adiposity**														
Waist to height ratio × 100	51.6	0.3	51.9	0.4	50.9	0.4	0.11	53.4	0.2	54.3	0.2	51.6	0.3	<0.0001
Waist to height ratio≥0.50	56.6%	1.7	60.2%	2.4	51.0%	2.2	0.006	69.6%	1.3	74.6%	1.6	58.8%	2.1	<0.0001
Waist to height ratio≥0.60	13.4%	1.1	14.1%	1.5	12.3%	1.7	0.41	15.6%	1.1	18.1%	1.5	10.4%	1.3	<0.0001

a Mean for interval variables, prevalence for binary variables (weighted estimates).

b Standard error of estimates taking sampling design into account.

c P-Value for Urban vs. Rural contrast, adjusted for sampling design.

**Figure 1:**
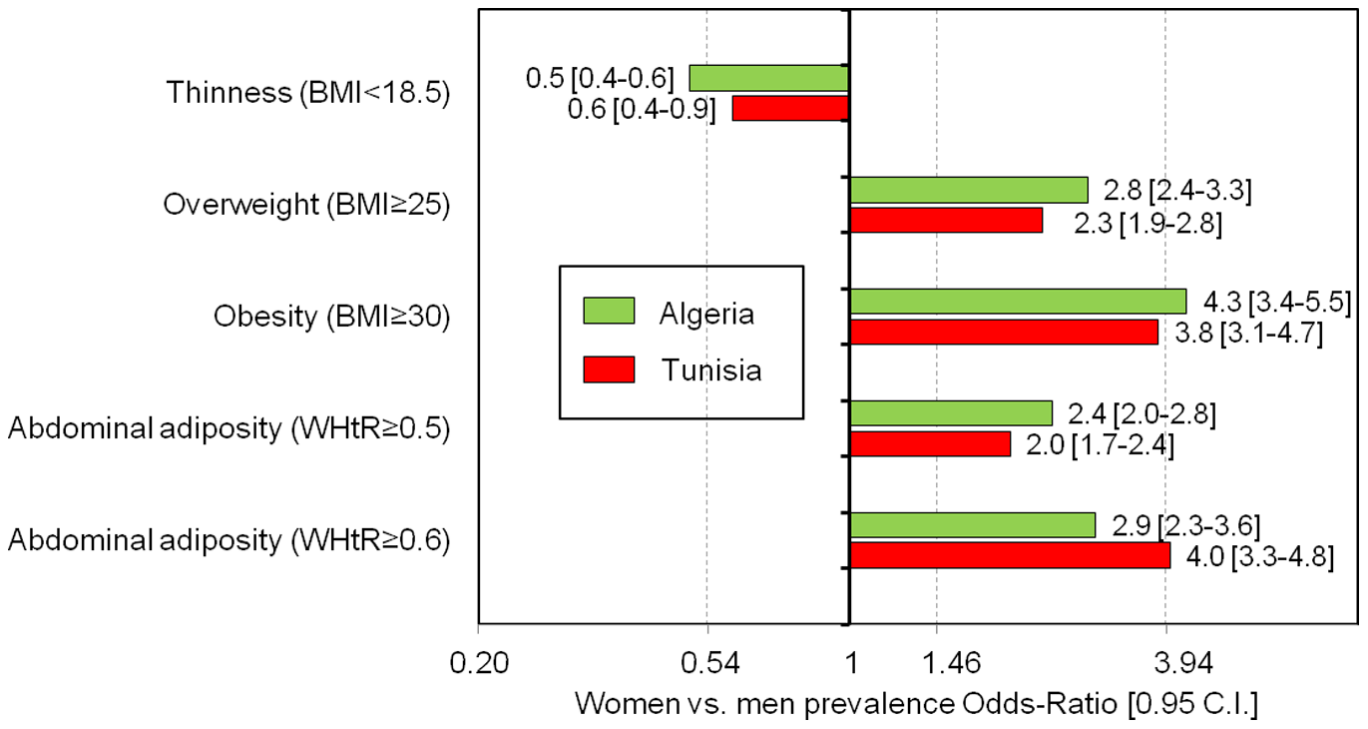
Gender contrasts of overall and abdominal adiposity among Algerian and Tunisian 35–70 year old adults. Women vs. men prevalence proportion odd-ratios weighted estimates for overall and abdominal adiposity, by country (Algeria women: n = 2742, men: n = 2004; Tunisia women: n = 2964, men: n = 2379). BMI: Body Mass Index in kg/m^2^, WHtR: Waist (cm) to Height (cm) Ratio. C.I.: 0.95 confidence interval adjusted for sampling design.

The gender inequalities regarding corpulence were high and quite similar in the two countries. The prevalence of thinness was much lower and the prevalence of overweight and obesity much higher among women versus men e.g. for obesity the women versus men OR was 4.3[3.4–5.5] (p<0.0001) in Algeria and 3.8[3.1–4.7] (p<0.0001) in Tunisia **(** **Table 2** **,** **Figure 1** **)**.

### Association of BMI and Obesity with Area of Residence and Socio-economic Factors

**Figure 2:**
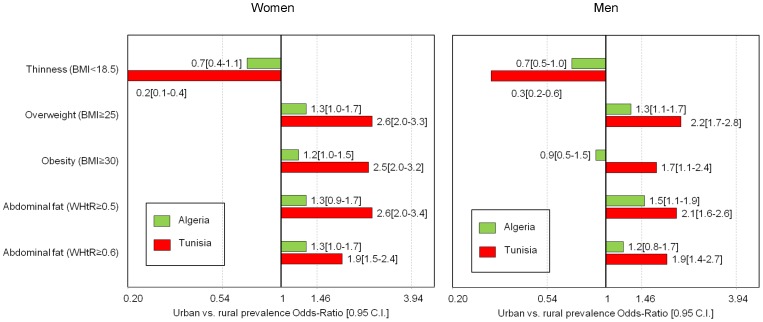
Urban vs. rural contrasts of overall and abdominal adiposity among Algerian and Tunisian 35–70 year old adults, by gender. Urban vs. rural prevalence proportion odd-ratios weighted estimates for overall and abdominal adiposity, by gender and country (Women Algeria: urban n = 1735, rural: n = 1007; women Tunisia: urban n = 1638, rural: n = 1326; men Algeria: urban n = 1185, rural: n = 819; men Tunisia: urban n = 1423, rural: n = 956). BMI: Body Mass Index in kg/m^2^, WHtR: Waist (cm) to Height (cm) Ratio. C.I.: 0.95 confidence interval adjusted for sampling design.

For most indicators and both genders **(** **Table 2** **,** **Figure 2** **)**, the urban versus rural contrasts were stronger in Tunisia than in Algeria, where they were mild for women and almost null for men. For example, for obesity (BMI> = 30): among women, the urban versus rural OR was 1.2[1.0–5.5] in Algeria compared to 2.4[1.9–3.1] in Tunisia and for men 0.8[0.5–1.4] in Algeria compared to 1.7[1.1–2.4] in Tunisia (detailed data not shown). After adjustment for demographic and socio-economic factors, the urban versus rural contrast persisted only among Tunisian women (OR = 1.4[1.1–1.8]) (detailed data not shown).

**Figure 3:**
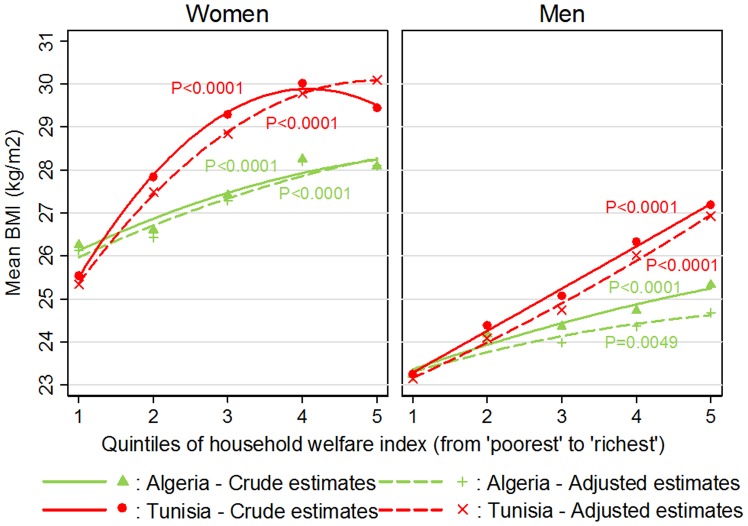
Association between the household welfare index and Body Mass Index among 35–70 year old Algerian and Tunisian adults, by gender. Association between quintiles of the asset-based household welfare index and Body Mass Index among Algerian and Tunisian adults, by gender. Comparison of unadjusted or adjusted (for age, marital status, education, and profession) BMI means by general linear models taking into account sampling design: P-value for null hypothesis of no difference in unadjusted or adjusted BMI means between quintiles of household welfare. Trends interpolated by quadratic fit. Complete case analysis (Algeria women: n = 2678, men: n = 1944, Tunisia women: n = 2725, men: n = 2238).

Among women in both countries **(** **Table 3** **)**, 45–54 year old subjects were more prone to obesity than other age groups, more so in Algeria. In Algeria the prevalence of obesity was higher among married than among single subjects, but not in Tunisia. The inverse U-shaped association of obesity with education (i.e. those who had only attended primary school were more prone to obesity) was more marked in Tunisia. As well, the association of obesity with professional occupation was stronger in Tunisia: after adjustment, women with an upper/intermediate-level profession were somewhat less prone to obesity than those who did not work outside the home. The increase in obesity **(** **Table 3** **)** or mean BMI **(** **Figure 3** **)** with the index of household welfare was much stronger in Tunisia than in Algeria. Moreover, in Algeria, the increase was essentially linear, while in Tunisia, there was a leveling off and/or a decreasing trend from the 4^th^ to the 5^th^ quintile.

**Table 3:** Association of obesity with socio-economic factors, among 35–70 year old Algerians and Tunisians, by gender.

	Obesity (BMI≥30 kg/m^2^)											
		Algeria						Tunisia				
		Unadjusted			Adjusted^a^			Unadjusted			Adjusted^a^	
	n	%^b^	OR^c^	C.I.^d^	OR^c^	C.I. d	n	%^b^	OR^c^	C.I.^d^	OR^c^	C.I. d
**Women**		**n** e ** = 2678**					**n** e ** = 2725**					
**Age**		P<0.0001			P = 0.0007			P = 0.018			P = 0.011	
35–44 y.	938	25.6	1	–	1	–	954	33.0	1	–	1	–
45–54 y.	914	36.2	1.6	1.4–2.0	1.5	1.2–1.9	951	41.8	1.5	1.1–1.9	1.5	1.2–2.0
55–70 y.	826	29.4	1.2	0.9–1.6	1.1	0.9–1.5	820	38.1	1.2	1.0–1.6	1.3	1.0–1.8
Marital status		P = 0.0008			P = 0.0026			P = 0.40			P = 0.34	
Married	2023	32.2	1.6	1.2–2.0	1.5	1.2–2.0	2175	37.6	1.1	0.9–1.4	1.1	0.9–1.5
Other	655	23.2	1	–	1	–	550	35.1	1	–	1	–
**Education**		P = 0.075			P = 0.36			P<0.0001			P = 0.0060	
No formal schooling	1496	29.5	1	–	1	–	1583	31.6	1	–	1	–
Primary	643	34.5	1.3	1.0–1.6	1.1	0.9–1.5	801	45.2	1.8	1.4–2.2	1.3	1.1–1.7
Secondary or more	539	26.8	0.9	0.7–1.2	0.9	0.6–1.2	341	38.0	1.3	1.0–1.8	0.9	0.6–1.3
**Professional occupation**		P = 0.042			P = 0.27			P = 0.53			P = 0.073	
Not working/retired	2396	30.9	1	–	1	–	2197	37.5	1	–	1	–
Employee/worker	145	22.9	0.7	0.4–1.0	0.8	0.5–1.3	405	38.1	1.0	0.7–1.4	1.2	0.8–1.6
Upper/intermediate	137	23.4	0.7	0.4–1.0	0.7	0.4–1.1	123	30.9	0.7	0.5–1.3	0.5	0.3–0.9
**Household welfare index^5^**		P = 0.014			P = 0.0049			P<0.0001			P<0.0001	
First quintile	634	23.4	1	–	1	–	747	16.7	1	–	1	–
Second quintile	497	27.6	1.2	0.9–1.8	1.2	0.9–1.7	677	33.1	2.5	1.8–3.5	2.4	1.7–3.3
Third quintile	486	29.1	1.3	1.0–1.9	1.4	1.0–1.9	581	45.0	4.1	3.0–5.6	3.9	2.8–5.3
Fourth quintile	558	34.4	1.7	1.2–2.4	1.8	1.3–2.5	401	47.9	4.6	3.3–6.4	4.6	3.2–6.6
Fifth quintile	503	34.3	1.7	1.2–2.5	1.9	1.3–2.8	319	46.3	4.3	2.9–6.4	5.7	3.7–8.6
**Men**	**n** e ** = 1944**						**n** e ** = 2238**					
**Age**		P = 0.74			P = 0.52			P = 0.89			P = 0.12	
35–44 y.	769	10.1	1	–	1	–	991	13.5	1	–	1	–
45–54 y.	528	8.0	0.8	0.4–1.5	0.8	0.4–1.5	672	14.4	1.1	0.7–1.6	1.1	0.7–1.7
55–70 y.	647	9.2	0.9	0.5–1.5	1.1	0.6–2.1	575	13.1	1.0	0.6–1.5	1.6	1.0–2.5
**Marital status**		P = 0.27			P = 0.28			P = 0.51			P = 0.69	
Married	1822	9.3	1.5	0.7–3.0	1.5	0.7–3.4	2156	13.5	0.7	0.3–1.8	0.8	0.3–2.0
Other	122	6.5	1	–	1	–	82	17.4	1	–	1	–
**Education**		P = 0.22			P = 0.36			P<0.0001			P = 0.021	
No formal schooling	580	7.2	1	–	1	–	506	6.0	1	–	1	–
Primary	602	9.4	1.3	0.9–2.1	1.3	0.9–2.0	916	14.5	2.6	1.6–4.3	2.1	1.3–3.8
Secondary or more	762	10.5	1.5	0.9–2.4	1.1	0.6–1.8	816	16.7	3.1	1.9–5.2	1.9	1.0–3.6
**Professional occupation**		P = 0.0029			P = 0.0035			P = 0.24			P = 0.083	
Not working/retired	648	8.5	1	–	1	–	400	10.4	1	–	1	–
Employee/worker	805	6.4	0.7	0.4–1.2	0.8	0.5–1.5	1349	13.9	0.9	0.6–1.2	1.6	1.0–2.5
Upper/intermediate	491	14.7	1.8	1.1–3.0	2.0	1.2–3.5	489	15.6	0.6	0.4–1.1	1.2	0.7–2.1
**Household welfare index** f		P = 0.076			P = 0.62			P<0.0001			P<0.0001	
First quintile	499	6.9	1	–	1	–	483	4.3	1	–		
Second quintile	406	7.8	1.1	0.5–2.4	1.1	0.5–2.3	481	10.7	2.7	1.4–5.1	2.3	1.2–4.3
Third quintile	368	10.5	1.6	0.9–2.7	1.4	0.8–2.5	478	13.1	3.4	1.9–6.0	2.9	1.6–5.1
Fourth quintile	352	9.3	1.4	0.8–2.5	1.2	0.7–2.2	455	17.6	4.8	2.7–8.5	4.0	2.2–7.4
Fifth quintile	319	11.8	1.8	1.1–3.0	1.4	0.8–2.6	341	21.3	6.0	3.4–10.8	5.5	2.9–10.3

a Adjusted for age, marital status, education, profession and household welfare index.

b Weighted proportion of obesity in each category.

c Prevalence Odds-Ratio vs. reference category.

d 0.95 design based confidence interval.

e Complete-case analysis.

f Asset-based household welfare index: increasing welfare from 1^st^ to 5^th^ quintile.

For men in both countries there was no association of age or of marital status with obesity **(** **Table 3** **).** Men with primary or secondary education were more obesity-prone in Tunisia only. Association with the occupational level was marked in Algeria where those with upper/intermediate professions were the most obesity-prone. In Tunisia, this association was weak. The association of the household welfare index with obesity or mean BMI **(** **Table 3** **,** **Figure 1** **)** was much flatter in Algeria than in Tunisia. In Tunisia there was a sharp linear increase in obesity and mean BMI with quintiles of the index so that about one fifth of the Tunisian men in the two top quintiles were obese.

## Discussion

In the context of the nutrition transition in North Africa, the present study assessed similarities and differences in corpulence and its relationships with gender, area of residence, and socio-economic factors between Tunisia and Algeria, based on large scale national data collected with similar methodology.

### Distribution of Corpulence in Algeria Compared to Tunisia

The prevalence of thinness was very low in Tunisia for both genders. However, it was not so for men in Algeria, among whom, even in this 35–70 year old age class, its prevalence requires specific attention and monitoring according to the World Health Organization (WHO) [28].

In both countries, two-thirds of the 35–70 year-old women were overweight and about one third were obese (i.e. similar or even higher prevalence than observed in some European countries [29]). Overweight and obesity are thus a major public health issue in both countries. There could be somewhat similar developmental origins to the right shift of the BMI distribution in both countries [3]. The history of nutrition over the life course, especially during the prenatal period, has been suggested as a likely factor of obesity, and it could be a significant factor in both Algeria and Tunisia among these subjects whose birthdates range from 1935 to 1970 [30]. However, the prevalences were higher in Tunisia, while Algeria was more similar to Morocco [13]. Men in both countries were also similar regarding overweight (about half the men) and obesity (about one-tenth), but the prevalences where also somewhat higher in Tunisia. Similar results were observed for abdominal obesity. This suggests a slightly more advanced nutrition transition in Tunisia, linked to a somewhat higher level of development [8] and to the associated changes regarding factors linked to the etiology of obesity, such as the availability and distribution of highly processed food [1], [31], [32]. Beyond the overall similarity discussed above, there could also be differences between the two countries regarding the developmental origins of obesity.

### Similarly High Gender Obesity Inequalities in the Two Countries

A marked similarity was that, contrary to what is observed in Europe [29], but in accordance with data in similar southern and eastern Mediterranean contexts [13], [33], women were much more overweight and obesity-prone than men in both countries. As a consequence, in both Algeria and Tunisia, women are to be considered a group especially at risk of obesity. On the other hand, this seems to protect them from under-nutrition as in Algeria only men featured a significant prevalence of thinness. Biological pathways, linked to the developmental origins of obesity, have been proposed to explain the higher prevalences of obesity in women versus men in developing countries [34], . As well, in the North African context, a common preference for plumpness in women is often suggested as a possible underlying cause [7], [34], [36], but this may be changing due to socio-economic development [37]. More importantly, the analogous gender obesity inequality in Algeria and Tunisia may mostly result from similarly non-egalitarian household and social roles of women versus men, with consequences for factors linked to the etiology of obesity such as sedentary behavior or increased food stimuli [34], [38], [39]. Tunisia is known to be one of the most progressive Arab countries regarding gender legislation and empowerment of women [10]. However, on a data-based international scale such as the Global Gender Gap Index, Algeria and Tunisia are quite close (respectively 119^th^ and 107^th^ out of 131 countries [9]).

### Urban versus Rural Corpulence Differential Higher in Tunisia

There was more thinness in rural areas in both countries: the higher prevalence was among rural men in Algeria where it bordered the 10% “medium prevalence/poor situation” WHO definition [28]. In both countries, urban subjects were more overweight or obese, but this was borderline in Algeria, while the urban versus rural contrast was much more marked in Tunisia. As in most LMIC, this was especially so for women [40], who were much more overweight and obesity-prone in urban than in rural areas in Tunisia (even after adjustment for socio-economic factors). This has been observed in the same Tunisian context [12], but not in Morocco [13], but we observed only a weak association in Algeria. The between-country differential regarding the urban-rural contrast was mostly due to urban subjects being more corpulent in Tunisia than in Algeria, while the rural areas were quite similar. This could be a marker of bigger differences in urban vs. rural lifestyles in Tunisia than Algeria, due to the more recent acceleration of urbanization in Algeria [41]. These results also differed from the observed trend of lower urban-rural differences in overweight, with an increase in the national prevalence of overweight, or in economic development [1], [40]. Tunisia, where the urban-rural contrast is higher, has nevertheless a slightly higher per capita gross domestic product (GDP) but also a generally higher level of economic development (with significant agricultural, mining, tourism, and manufacturing sectors) than Algeria, whose GDP relies heavily on the oil and natural gas rent [42]. There could also be comparability issues regarding urban versus rural categorization in each country [12], [43].

### Association with Socio-economic Position More Marked in Tunisia

There were few observed associations of thinness with age or socio-economic factors in Algeria except for a slightly higher prevalence among men from the lowest quintile of economic welfare (detailed data not shown). For men, professional occupation was more clearly linked to obesity in Algeria as the upper/intermediate professions were more obesity prone than the others, while this was only (more mildly) so in employee/workers in Tunisia. Otherwise, the associations between corpulence and indicators of SEP were more marked in Tunisia. Those of primary or secondary education were twice as prone to obesity, and there was a sharp linear increase in obesity and/or mean BMI with quintiles of the household welfare index in Tunisia, but much less so in Algeria. Comparison with other studies was difficult due to different measurements or categorization (e.g. in Morocco [13]), or simply because many cross-country studies only focus on women [44].

In Algeria, married women were more obesity prone, as has also been observed in Morocco [13], [34], but not in Tunisia as also previously observed [12]. There have been conflicting results regarding this association [45]. But it should be noted that, in the studied age class, both in Algeria and Tunisia, the not married women are mostly divorced women **(** **Table 1** **)**. Although the law allows women to divorce in both countries, in such socio-cultural contexts, being a divorced women is not socially well-accepted [46]. Nevertheless, there are variations around that general trend. In Tunisia, women’s status (either regarding legislation or social role) is more in emancipated than in Algeria [10], [47]. So it is consistent that the difference in obesity rates between married and divorced women is the lowest in the country where being divorced is socially more well-accepted. It remains for future work to explain why, in Algeria, even after adjustment for socio-economic factors, divorced women were actually less prone to obesity than married ones.

No association was found between education and obesity in Algeria. Similarly, a study in Morocco found no association between obesity and education among women over 18 years in age [13]. In Tunisia, obesity was more frequent among women with primary education than among those with no education or with secondary education or more, as also found in previous studies [12]. These women could be more exposed than illiterate women to obesogenic lifestyle factors such as a high energy intake and a sedentary lifestyle, without the moderating factors such as better perception of etiology of obesity or slimmer body image norms of better educated women [48]. Association of professional activity with obesity was also observed only in Tunisia: there, women with upper/intermediate professions were less obesity prone, possibly due to a combination of factors such as the symbolic value of a slimmer body, having a healthy lifestyle and differential household and social role compared to those in lower professional categories [4], [48]. The prevalence of obesity and the mean BMI increased with quintiles of the household asset-based welfare index in both countries, but the association was much stronger in Tunisia than in Algeria. Also, prior to adjustment for other socio-economic factors, there was a flattening of the curve and even a decrease in the last two quintiles in Tunisia, likely because the intrinsically obesogenic environment of a higher welfare index household (as assessed by the adjusted association) was moderated by higher levels of other indicators such as education or profession. There was no such curvature in Algeria, as the increase was basically linear as observed in many LMIC (although not all, e.g. Egypt, Bolivia) [44] and also much flatter than in Tunisia, similar to the relationship between declared income and obesity among women in Morocco [13].

Generally, our results are in line with those observed in LMIC where within-country there is an increase of obesity rates with household welfare, while on the contrary, within developed countries the burden of obesity is heavier among the poorest [44]. Nevertheless, even in the lowest quintile of the household welfare index, a fifth of the women were obese in both Algeria and Tunisia so that obesity is already a public health issue even among the lower economic welfare households in both countries [49]. However, this is not exclusive of a situation of double burden of malnutrition at country-level as in Algeria, where the prevalence of thinness among men is still significant especially in rural areas. This double burden of overweight/obesity and thinness has been reported in other LMIC either at community [50] or household level [51]. As often reported, the links between indicators of socio-economic position and corpulence were also weaker among men than women [4]. Nevertheless, our results suggest for both genders, even if more so for women, a stronger socio-economic patterning of corpulence and obesity in Tunisia. The patterning was less marked in Algeria and more similar to what is observed in most LMIC. Apart from possible comparability issues regarding measurement of socio-economic position [6], and in addition to very different political and societal histories in the last two decades [11], the difference could have several root causes. These causes may include a quite different pattern of economic development policy in recent decades: Tunisia has a generally more market oriented economy whereas the economy was more dominated by the state in Algeria. Also, as already mentioned, Tunisia has a more diversified economy than Algeria which is largely dependent on hydrocarbon rent. Tunisia consequently also had a somewhat higher income inequality (as assessed by the Gini concentration Index) than Algeria (63^rd^ versus 89^th^ out of 140 countries in decreasing order of inequality) [42]. These differences in types of economic development could have resulted in a higher variability with socio-economic status in Tunisia than Algeria of obesity related lifestyle factors such as food consumption and physical activity (as we also discussed it for urban-rural contrasts). The difference between Algeria and Tunisia in the relationship between corpulence and socio-economic factors could also be due to country-specific interactions between developmental origins of obesity and those lifestyle factors [3].

### Methodological Strengths and Limitations of the Study

The cross sectional design has limitations regarding causal interpretation and also does not account for time trends. A major strength was our large national samples, resulting from comparable surveys performed at the same time period, with a similar survey design, questionnaire, data collection protocol and analyses in Algeria and Tunisia. In particular, we added to the available epidemiological data, especially given the scarcity of large-scale epidemiological data in Algeria, with data for both genders. As for the target population of 35–70 year old adults, though this age class was used in a number of studies pertaining to risk factors of chronic diseases [16], the lack of younger adults is a limit of the study. Comparability of the two countries regarding measurements of the urban versus rural environment [12], [43], the coding of professional activity, and the ranking of households on the welfare index could be an issue. As for the welfare index, conditional on a relevant choice of items in each country, the within-country ranking of households and thus relative interpretation of the quintiles would be comparable between countries [6]. As well, using different dimensions of socio-economic position such as education, profession and household welfare likely minimized most problems of between-country comparability. We did not discuss data on intermediate risk factors of obesity such as physical activity or dietary intake but these topics were beyond the scope of the paper which focused on between-country differences in the relationship of obesity with more distal socio-economic factors.

## Conclusion

The aim of the present study was to compare Algeria and Tunisia regarding the prevalence of obesity and its associated socio-economic factors, in the context of the large-scale trends of globalization, modernization and societal changes that both countries are currently experiencing [1]. The nutrition transition appears more advanced in Tunisia: while both countries showed a high prevalence of overweight and obesity (especially among women), the overall burden of overweight and obesity was somewhat higher in Tunisia. As well, in Algeria there was to some extent a double burden of malnutrition [52] at the national level, because thinness was persistent among men. We showed a similar high gender obesity gap in both countries, as women were much more corpulent than men. This was likely due to the similar socio-cultural gender-related issues common to countries in the region, although differences exist between the two countries in the relationship between marital status and obesity. We also highlighted country-specific environmental and socio-economic patterning of corpulence by showing that the relationship with area of residence (urban/rural) and household welfare was much more marked in Tunisia. Nevertheless in both countries even among the poorest households, a fifth of the women were obese. Taking into account the developmental origins of obesity, this could foreshadow an even higher burden of obesity among the poorest in the subsequent generations, in turn fuelling even larger inequalities in health [3]. The observed similarities and differences should be taken into consideration for managing the heavy burden of overweight, obesity and related NCDs in the two countries. Prevention of obesity, especially among women, is a major public health issue in both Tunisia and Algeria, though with country-specific issues regarding the management of obesity inequalities (here associated with area of residence or socio-economic position) that should be taken into account when devising national obesity policies [53].
